# Role of HIV exposure and infection in relation to neonatal GBS disease and rectovaginal GBS carriage: a systematic review and meta-analysis

**DOI:** 10.1038/s41598-017-13218-1

**Published:** 2017-10-23

**Authors:** Piet Cools, Janneke H. H. M. van de Wijgert, Vicky Jespers, Tania Crucitti, Eduard J. Sanders, Hans Verstraelen, Mario Vaneechoutte

**Affiliations:** 1 Laboratory Bacteriology Research, Department of Microbiology, Immunology and Clinical Chemistry, Faculty of Medicine and Health Sciences, Ghent University, De Pintelaan 185, Ghent, Belgium; 2 Department of Clinical Infection, Microbiology and Immunology, Institute of Infection and Global Health, University of Liverpool, Brownlow Hill, Liverpool, UK; 3 HIV and Sexual Health Group, Department of Public Health, Institute of Tropical Medicine, Nationalestraat 155, Antwerp, Belgium; 4 HIV/STI Reference Laboratory, Department of Clinical Sciences, Institute of Tropical Medicine, Nationalestraat 155, Antwerp, Belgium; 5 Centre for Geographic Medicine Research - Coast, Kenya Medical Research Institute (KEMRI), PO Box 230-80108 Kilifi, Kenya; 6 Nuffield Department of Medicine, University of Oxford, Headington, United Kingdom; 7 Department of Obstetrics and Gynaecology, Vulvovaginal Disease Clinic, Ghent University Hospital, De Pintelaan 185, Ghent, Belgium

## Abstract

*Streptococcus agalactiae* (GBS) is the leading cause worldwide of neonatal sepsis. We sought to assess to which extent HIV exposure of neonates is associated with GBS neonatal disease. Furthermore, we assessed to which extent HIV infection in women is associated with maternal rectovaginal GBS carriage, the single most important risk factor for GBS neonatal disease. We searched Pubmed, Embase, and Web of Science for studies assessing the association between neonatal GBS disease and HIV-status of the mother and studies that assessed the association between rectovaginal GBS colonization and HIV status in women. HIV-exposed uninfected neonates were more than twice as likely to have neonatal GBS disease compared to unexposed neonates. HIV-exposed neonates were not at increased risk for early-onset neonatal disease, but were 4.43 times more likely to have late-onset neonatal GBS disease. There was no significant association between HIV infection status and rectovaginal GBS carriage. Public health interventions preventing neonatal GBS disease are urgently needed for the increasing group of HIV-exposed neonates. A framework integrating and explaining our findings highlights opportunities for the clinical practice and global health policy to prevent disease. Well-designed studies should clarify the relation between HIV-status and GBS carriage.

## Introduction

One million children die each year in the first 4 weeks of life because of neonatal disease^1^. Early-onset neonatal disease (EOD), which occurs in the first week of life and most often within 24 hours after birth, is caused by bacteria that are transmitted from the genital tract of the mother before or during delivery^2^. Late-onset neonatal disease (LOD) occurs between the first week and the third month of life and may be caused by bacteria acquired vertically or horizontally^3^.

*Streptococcus agalactiae* (Group B *Streptococcus* (GBS)), present in the vagina and/or gastro-intestinal tract of 20 to 30% of pregnant women, is the most common infectious agent identified in case of EOD^4^ and an important pathogen in LOD^5^. Although vaginal carriage of GBS usually remains asymptomatic, it has been associated with chorioamnionitis^6^, spontaneous abortion^7,8^, premature labor^9^, premature delivery^10^, premature rupture of membranes^11^ and puerperal sepsis^12,13^, causing substantial neonatal and maternal morbidity and mortality^1,2,14,15^.

Infants born to HIV-infected mothers have increased rates of infectious morbidity and mortality compared to non-exposed infants, even if they remain HIV-uninfected^16,17^. Since the first study reporting an increased risk of GBS neonatal disease in HIV-exposed neonates^17^, evidence for this association has been accumulating.

A possible explanation for these observations might be higher rectovaginal GBS carriage rates in HIV-infected women, as rectovaginal GBS carriage is a major risk factor for GBS neonatal sepsis.

Indeed, HIV might influence the microbial composition of the vagina, rectum and colon^18–22^ and HIV infection in women has been associated with increased prevalence of vaginal candidiasis and sexually transmitted infections^23,24^. However, conflicting results have been reported for the association between HIV infection and rectovaginal carriage of GBS^25,26^.

The objectives of this systematic review and meta-analysis were to assess to which extent HIV exposure of neonates is associated with GBS neonatal disease and to clarify to which extent HIV infection of women is associated with rectovaginal GBS carriage, a major risk factor for GBS neonatal disease.

## Research in context

### Evidence before this study

Mother-to-child transmission of HIV is one of the great tragedies of the HIV pandemic. Although the number of HIV-infected infants is declining due to interventions for the elimination of pediatric HIV infection, the number of newborns that are HIV-uninfected but HIV-exposed through their HIV-infected mothers is on the rise. Interest in the health outcomes of these HIV-exposed infants has grown in the past decade, and several studies suggest increased mortality rates and increased infectious morbidity.

Neonatal sepsis causes one million neonatal deaths yearly, and Group B *Streptococcus* (GBS) is the leading cause. Since 2010, evidence has also been accumulating for an association between HIV-uninfected neonates born to HIV-infected mothers and neonatal sepsis caused by Group B *Streptococcus* (GBS). Regarding the association between maternal vaginal GBS carriage – the single most important risk factor, if not, a prerequisite, for GBS neonatal sepsis – and HIV-infection in mothers, conflicting results have been reported.

### Added value of this study

To our knowledge, this systematic review and meta-analysis is the first to examine the association between HIV-exposure of neonates and the risk for neonatal disease caused by GBS. We performed subgroup analysis to further clarify the risk for the early-onset form of GBS neonatal sepsis, and the late-onset form. Furthermore, we performed a meta-analysis to assess the risk for maternal vaginal GBS carriage in HIV-infected compared to uninfected women. We present a pathogenic framework, explaining our findings regarding the associations between neonatal HIV-exposure and early-and late-onset neonatal sepsis. This framework offers opportunities for the clinical practice and global health policy to reduce HIV-related neonatal morbidity and mortality rates.

### Implications of all the available evidence

Neonates born to HIV-infected mothers, but themselves uninfected, are at increased risk for GBS neonatal sepsis. Subgroup analysis showed that these neonates were four times more likely to have the GBS late-onset form, but were not at increased risk for the early-onset form. We found no association between maternal vaginal GBS carriage and HIV-infection, but included studies suffered from major limitations.

The health needs of HIV-exposed, uninfected infants should be prioritized further. Particularly in sub-Saharan Africa, were 90% of new HIV infections occurs and the burden of neonatal mortality is the highest, public health interventions reducing neonatal mortality rates are urgently needed. Feeding strategies should be explored to reduce LOD in infants born to HIV-infected mothers.

## Methods

### Protocol and registration

We followed the Preferred Reporting Items for Systematic Reviews and Meta-Analyses (PRISMA) guidelines^27^ for carrying out and reporting this systematic review and meta-analysis, using an *a priori* defined protocol. This systematic review and meta-analysis has been registered in PROSPERO under registration number CRD42016033414.

### Eligibility criteria

We included studies that assessed the association between neonatal GBS disease and HIV-status of the mother and studies that assessed the association between rectovaginal GBS colonization and HIV status in adult women. We only included studies that reported a sample size and a measure of effect for the associations assessed or provided the data to calculate the latter. There were no restrictions in terms of study design, manuscript language, or date of publication. We included papers that were published up to 1 November 2015.

Reviews, conference abstracts, comments, guidelines, case reports or case series, unpublished articles, multiple reports of the same data and *in vitro* and animal studies were not considered for inclusion. We excluded studies that had no clear description of the assessment of the HIV status of the women, of the HIV exposure of the neonates, or of the laboratory methodology to assess rectovaginal GBS carriage. GBS neonatal disease was defined as the presence of GBS as assessed by molecular methods, culture from blood, cerebrospinal fluid or another normally sterile site, or by latex agglutination in cerebrospinal fluid, in neonates below 90 days of age.

### Search strategy

We searched the MEDLINE (through PubMed), Web of Science, and EMBASE databases for bibliographic references. We used the combination of all of the following search terms (*Streptococcus agalactiae*, *S. agalactiae*, GBS, Group B *Streptococcus*, Group B streptococci, early-onset disease, early-onset sepsis, late-onset disease, late-onset sepsis, neonatal sepsis, neonatal septicemia, neonatal pneumonia or neonatal meningitis) with all of the following search terms (human immunodeficiency virus, HIV, HIV-1, or AIDS).

The bibliographic references of the studies that were considered eligible were also hand searched.

### Study selection

**Figure 1:**
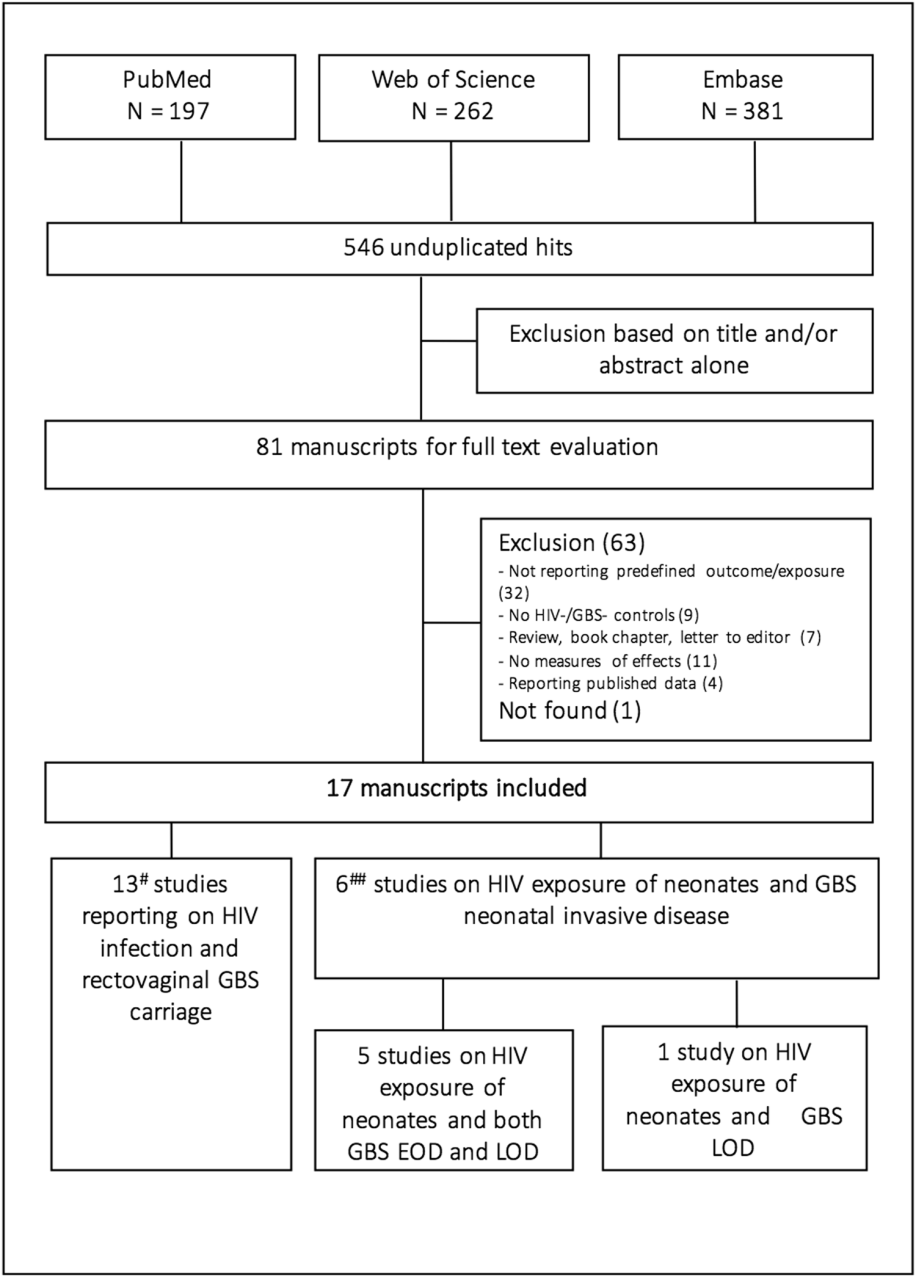
Identification of studies included in the meta-analysis. ^#^Including two studies reporting on neonatal GBS disease; ^##^including two studies reporting on rectovaginal carriage.

Studies were selected in a two-stage process (Fig. 1). First, all bibliographic references were screened to identify studies for full text evaluation by one reviewer (PC), on the basis of only the information present in the title and abstract. Subsequently, the full texts of those articles not excluded in the screening process were assessed for eligibility, using the aforementioned criteria, by two authors independently (PC and MV). Reasons for inclusion or exclusion were recorded and categorized, and disagreements were resolved by consensus (Supplementary Table 1).

Selected studies were divided into two categories, i.e. studies reporting an association between HIV-exposure and GBS invasive neonatal disease in infants (or providing data to calculate this), and studies reporting an association between HIV infection and rectovaginal GBS carriage in adult women (or providing data to calculate this).

### Data collection process

A predefined data extraction form was pilot-tested and used for data extraction. From each individual study, information was collected on authors, year of publication, journal, country where the study had been carried out, study design, sample collection period, study population, age of the participants, total number of study participants, total number of samples included in the analysis, HIV assessment and prevalence, GBS neonatal disease assessment and incidence, rectovaginal GBS carriage assessment and prevalence, relevant measures of effects, statistics, correction for confounding, and epidemiological strengths and weaknesses.

This procedure was performed for each article independently by two authors (PC, and TC, VJ, ES, JVDW or MV) and disagreements were resolved by discussion between both authors. Further information or clarification from manuscript authors was sought by PC on seven occasions.

### Risk of bias in individual studies

To assess the quality and risk of bias of the included studies, we developed a quality appraisal tool (Supplementary Table 2), based on the Newcastle-Ottawa Scale^28^. This scale is a bias assessment tool for observational studies, recommended by the Cochrane Collaboration^29^. We adapted this tool to the content of the meta-analysis. For example, consideration was given to the methodology used to assess rectovaginal GBS carriage.

Stars were assigned by PC for three broad criteria (representativeness of the study groups, comparability of the study groups, and quality of the outcome assessment) in order to provide a measure of their quality. In this context, good representativeness is defined as those exposed/unexposed being similar to others in the community they come from, and good comparability is defined as the exposed and unexposed being similar in all respects other than their HIV or HIV exposure status.

Studies that scored none or one, two or three, or four stars for representativeness were classified as having respectively a high, medium or low risk of bias for this criterion. Studies assigned none, one, or two stars for comparability or outcome were considered to have respectively a high, medium or low risk of bias for comparability and outcome.

### Data analysis

First, an odds ratio (OR) was calculated for studies that did not present them. We compared the odds of neonatal GBS disease in neonates born to HIV-infected mothers versus infants born to HIV-uninfected mothers, and the odds of rectovaginal GBS carriage in HIV-positive versus HIV-negative women, using unadjusted counts. All neonates born from HIV-positive women were considered as HIV-exposed, regardless of their infection status. Rectovaginal GBS carriage was defined as GBS detected in the vagina and/or rectum and/or (peri)anal region by culture. While our protocol also included detection of rectovaginal and neonatal GBS by molecular testing, none of the eligible studies used molecular testing, and the results reported in this manuscript are based on culture and latex agglutination results only.

We reported the results of the meta-analyses obtained after pooling individual study estimates with random effects model as ORs with 95% confidence interval (CI). The degree of heterogeneity between the studies was assessed using the I^2^ index, with percentages of 30–50%, 50–75% and 75–100% being indicative of moderate, substantial and considerable heterogeneity, respectively^30^.

A priori planned subanalyses were performed for GBS neonatal disease by considering early-onset and late-onset neonatal GBS disease separately, relying on the authors’ own classification into these two groups.

To assess publication bias, funnel plots were created by plotting the natural logarithm of the ORs against the inverse of the standard error. The asymmetry of the funnel plots was visually inspected and statistically checked using Egger’s regression test and Begg and Mazumdar’s rank correlation test^31,32^. All statistical analyses were carried out using the Comprehensive Meta-Analysis software package v3 (Biostat Inc., Englewood, NJ).

### Role of the funding source

The funder of the study had no role in study design, data collection, data analysis, data interpretation, or writing of the report.

## Results

### Manuscript and study selection

A total of 546 unduplicated citations were identified, we selected 81 abstracts for full text evaluation (Fig. 1). Of these, 32 manuscripts did not report results on GBS disease or carriage or HIV exposure, seven were reviews, book chapters or letter to editors, nine did not include an HIV-negative or GBS-negative group, 11 did not present measures of effect or the data to calculate these, four reported already published data, and one paper could not be found (Supplementary Table 1).

Seventeen manuscripts reporting on 19 studies met the inclusion criteria. These included four studies reporting on neonatal GBS disease only, 11 studies reporting on rectovaginal carriage only, and two studies reporting both (Fig. 1). A list of these studies and a digest of the extracted information is presented in the Supplementary Tables 3 and 4.

### Description of selected studies

Six studies, reporting a total of 252,051 births, reported cases of infants with GBS neonatal disease born to HIV-infected mothers compared to infants with GBS neonatal disease born to HIV-uninfected mothers^17,33–37^. Five of these studies were conducted in South Africa^33–37^ and one was performed in Belgium^17^. All infants included were born in a hospital. For the laboratory diagnosis of GBS neonatal disease, all studies used the BacT/ALERT microbial detection system to detect bacteria in blood and/or cerebrospinal fluid samples. Positive samples were subcultured on blood agar plates and identification was subsequently performed using standard identification methods.

For the second category, thirteen studies, representing a total of 10,105 women, provided rectovaginal GBS carriage ratios of HIV-infected versus HIV-uninfected women^7,25,26,34,35,38–45^. Eleven of these studies were conducted in low-or-middle income countries, i.e. three studies in South Africa^34,35,43^, two studies in Tanzania^39,41^, and one study in Burkina Faso, Brazil, Democratic Republic Congo, Kenya, Malawi, and Zimbabwe^7,25,26,38,40,42^ and two in a high-income country namely Spain and the US^44,45^. All these studies recruited pregnant women in a hospital setting, except for the study of Djigma and coworkers in Burkina Faso (2011), who recruited non-pregnant women, and the study of Ulla and coworkers in Spain (1993), who recruited female sex workers attending a family planning center.

Rectovaginal GBS carriage was assessed by culture in all studies: five studies performed rectovaginal sampling and culturing with the use of an enrichment broth, as recommended by the Centers for Disease Control and Prevention (CDC) guidelines^26,40–42,44^, two studies only sampled vaginally with cultures performed according to the CDC guidelines^34,35^ and the remaining six studies did not culture according to CDC guidelines or did not report the exact culture methods used^7,25,38,39,43,45^.

Of the 19 studies described of the 17 manuscripts, three were retrospective^17,37,44^, fourteen were cross-sectional^7,25,26,33–36,39–43^ and none were prospective.

One study reported two different analyses for rectovaginal GBS carriage in HIV-infected versus uninfected women, i.e. using the total cohort versus matched subsets^35^. The analysis least prone to bias (i.e. the matched subsets) was used for this meta-analysis. The same study analyzed matched subsets for the association of HIV exposure of neonates and GBS neonatal disease and early-onset GBS neonatal disease, but not for late-onset GBS neonatal disease. Therefore, we used the total cohort analysis for these associations.

### Risk of bias within studies

**Figure 2:**
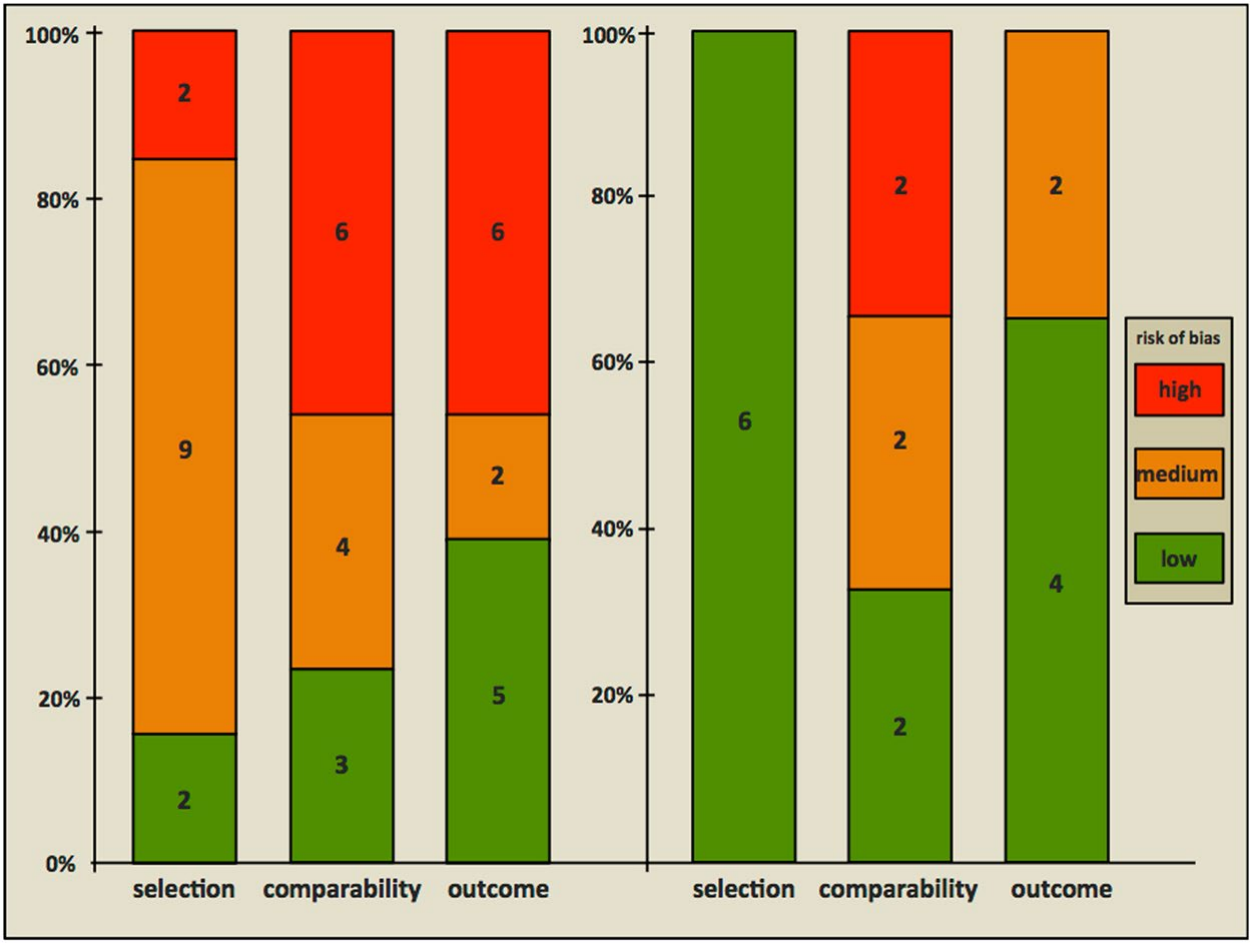
Quality assessment using an adapted Newcastle-Ottawa Scale for risk of bias of studies included in the systematic review. Left, studies (n = 13) reporting an association between HIV infection and rectovaginal GBS carriage. Right, studies (n = 6) reporting an association between neonatal HIV exposure and neonatal GBS disease. The absolute numbers of studies are shown in the boxes.

The results of the critical appraisals of the included studies are shown in Fig. 2 and Supplementary Table 5. All six studies addressing the relationship between HIV exposure of neonates and GBS neonatal disease were assessed as having a low risk of bias for representativeness^17,33–37^. Two out of the six studies had a low risk of bias for comparability^33,35^, two had a medium risk^34,36^ and two had a high risk^17,37^. For the GBS disease outcome assessment, four studies were assessed as having a low risk of bias^33,35–37^ and two had a medium risk^17,34^.

Of the studies on HIV infection and maternal GBS carriage only two out of the thirteen studies were assessed as having a low risk of bias for representativeness^35,40^, nine studies a medium risk^7,25,26,34,39,41–43,45^ and two studies a high risk^38,44^.

Three out of the thirteen studies were assessed as having a low risk of bias for comparability^35,40,44^, four a medium risk^34,39,41,43^, and six a high risk^7,25,26,38,42,45^. The risk of bias for outcome assessment was low in five out of the thirteen studies^26,40–42,44^; medium in two studies^34,35^ and high in six studies^7,25,38,39,43,45^.

### Association of maternal HIV status with GBS neonatal disease

**Figure 3:**
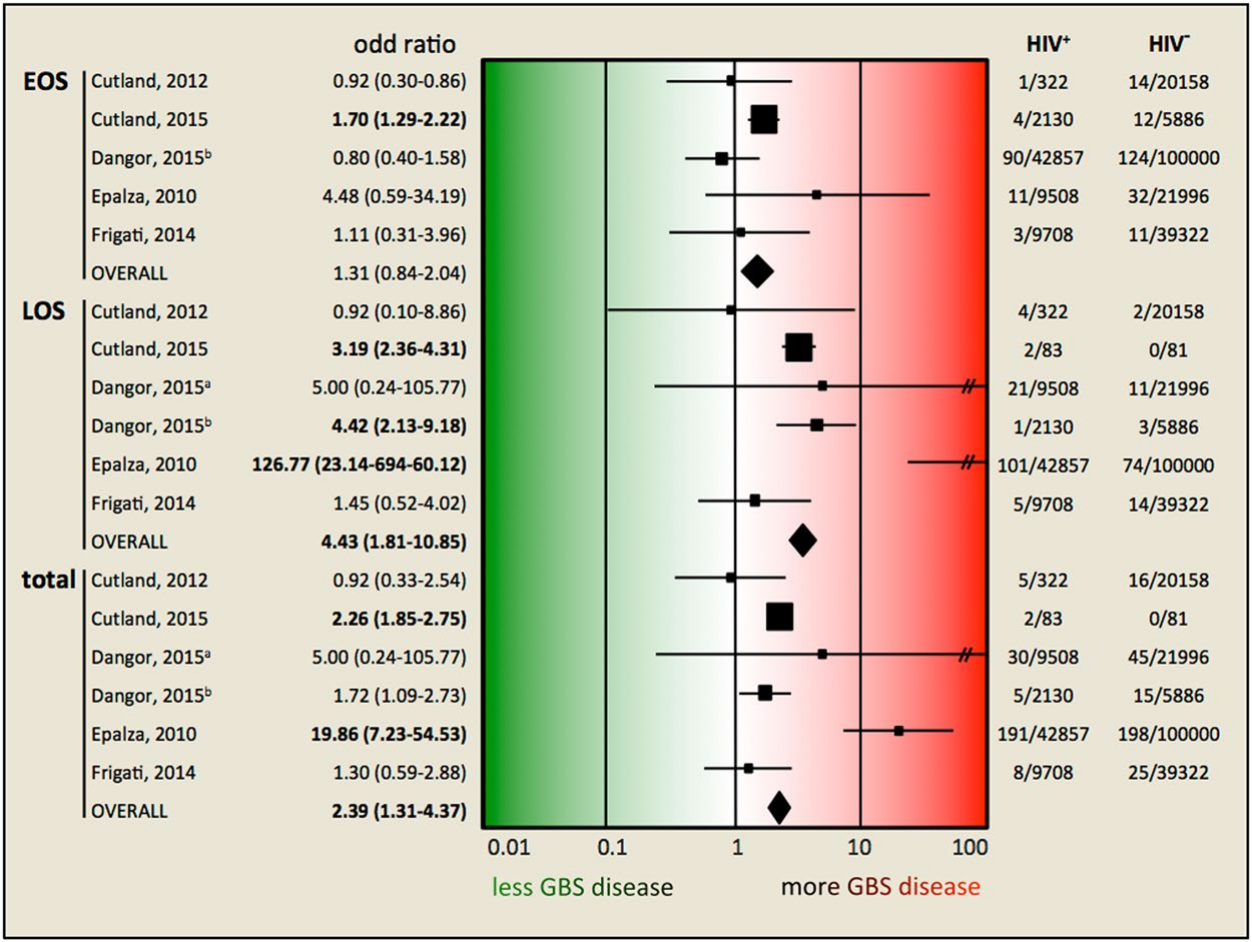
Forest plot of estimates of association between HIV exposure and GBS neonatal disease. Studies are plotted alphabetically according to the last name of the first author and followed by the publication year. Each study is represented by a black square and a horizontal line, which corresponds to the OR and 95% CI, respectively. The areas of the black squares reflect the weight of the study (determined by random effects analysis) in the meta-analysis. The vertical line in the middle corresponds to an OR of 1.0. The diamonds represents the overall summary estimate for EOD, LOD and total GBS disease, respectively, with the 95% CIs given by the width. In the columns on the right, the number of cases on the total number of HIV-positive and of HIV-negative mothers is given, respectively, for each study. ^a^Dangor, 2015, JID; ^b^Dangor, 2015, PONE; CI, confidence interval; OR, odds ratio; //95% CI line exceeding OR 100.

The meta-analysis of the association between HIV exposure of neonates and GBS neonatal disease showed that HIV-exposed neonates were more than twice as likely to have neonatal GBS disease compared to unexposed neonates (OR, 2.39; CI, 1.31–4.38; p = 0.005) (Fig. 3). The crude OR in the individual studies ranged from 1.72 to 19.86, with three of the six individual studies showing a statistically significant association. The subanalyses showed that HIV-exposed neonates were not at increased risk for early-onset neonatal disease (OR, 1.31; 95% CI, 0.84–2.04; p = 0.240), but were 4.43 times more likely to have late-onset neonatal disease (95% CI, 1.81–10.85; p = 0.001).

As recommended by Sterne and co-workers (2011)^46^ to only create funnel plots when the number of studies is ten or more, we did not create a funnel plot for this meta-analysis.

### Association of HIV infection and rectovaginal GBS carriage

**Figure 4:**
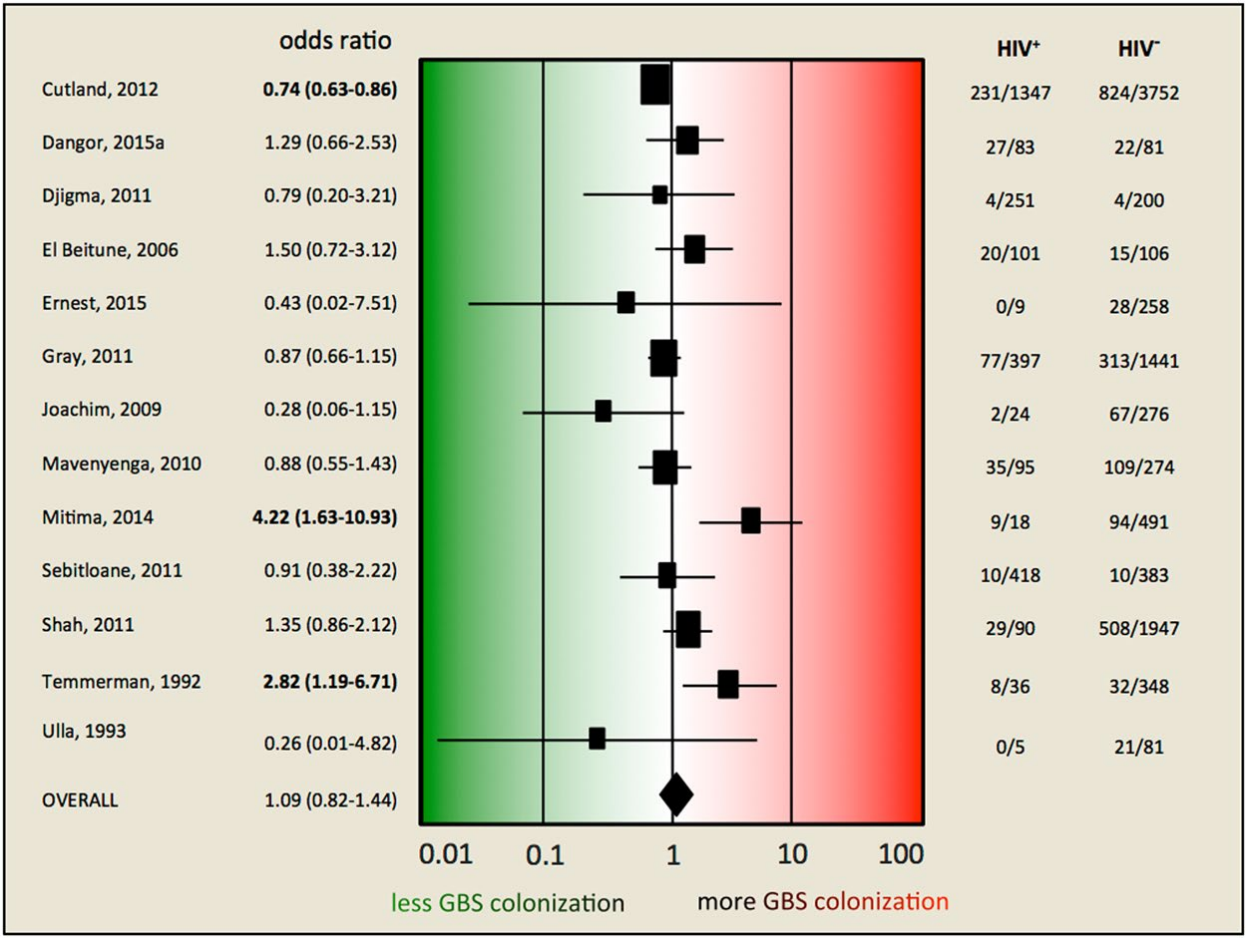
Forest plot of estimates of association between HIV infection and rectovaginal GBS carriage. Studies are plotted alphabetically according to the last name of the first author and followed by the publication year. Each study is represented by a square and a horizontal line, which corresponds to the OR and the 95% CI, respectively. The magnitude of the squares reflects the weight of the study (determined by random effects analysis) in the meta-analysis. The vertical line in the middle indicates the OR 1.0. The diamond at the bottom represents the summary estimate, with its width indicating the 95% CI. The two columns on the right document the number of GBS cases on the total of HIV-positive and of HIV-negative women, respectively, for each study. ^a^Dangor, 2015, JID; CI, confidence interval; OR, odds ratio; bold, p < 0.05.

In our meta-analysis of the association between HIV infection and rectovaginal GBS carriage, we found no significant association between HIV infection status and rectovaginal GBS carriage (OR 1.09; 95% CI 0.82–1.44; p = 0.55) (Fig. 4). The crude ORs in the individual studies ranged from 0.26 to 4.22, with only three studies reporting a statistically significant association^7,25,35^. Two of these studies found a significantly higher rectovaginal carriage rate in HIV-positive women^7,25^, whereas one study found a significantly lower rate^35^.

**Figure 5:**
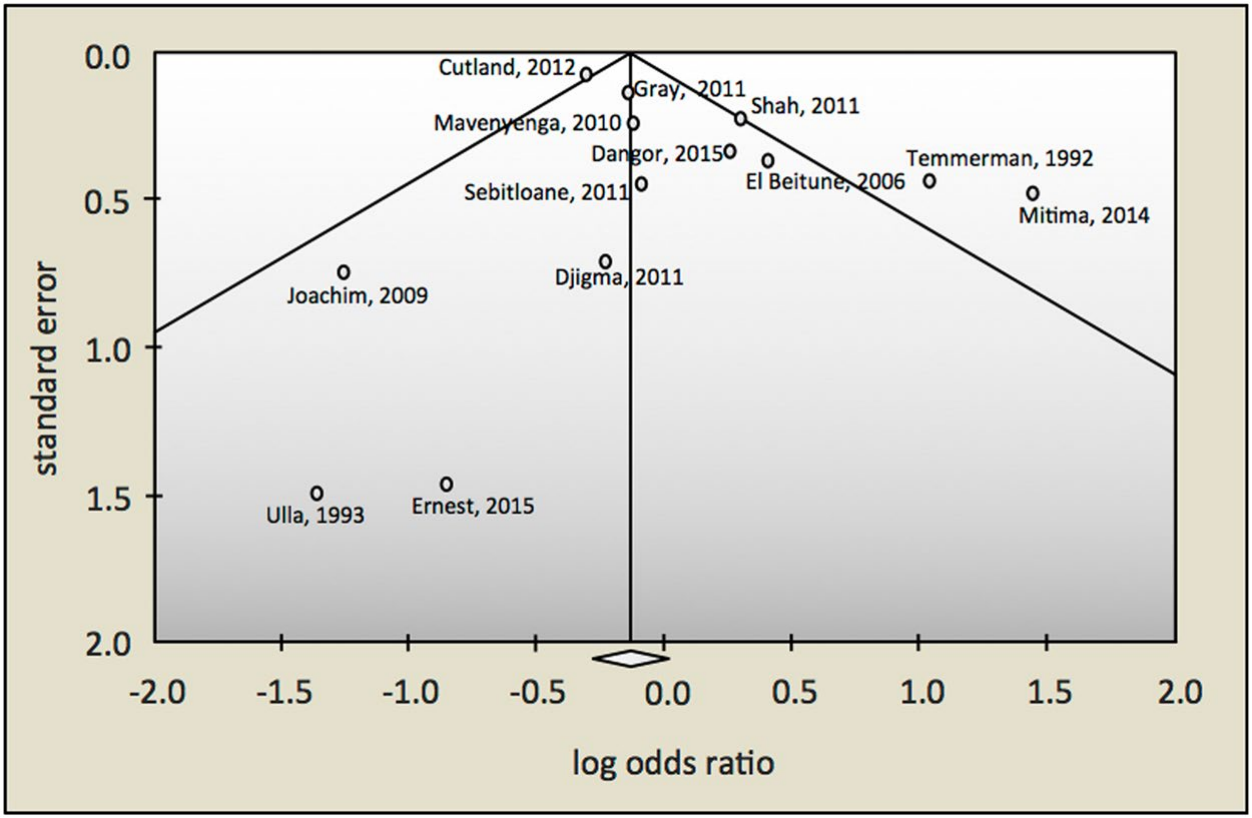
Funnel plot to assess publication bias among studies evaluating the association between HIV infection and rectovaginal GBS carriage. The circles represent the estimates of the 11 included studies of association between HIV infection and rectovaginal GBS carriage. The log of the odds ratio is plotted on the horizontal axis, against the inverse of the standard error of the odds ratio on the vertical axis. The vertical line in the funnel plot indicates the fixed-effects summary estimate and the sloping lines indicate the expected 95% confidence intervals for a given standard error.

The heterogeneity among these studies is substantial (I^2^ = 68.7%). Visual inspection of the funnel plot shows an asymmetrical distribution of studies, suggesting publication bias (Fig. 5), which is marginally supported by Egger’s regression intercept (0.94; 95% CI −0.48–2.37; p = 0.09), but not by the Begg and Mazumdar’s rank correlation test (Kendall’s tau = −0.10, one-tailed p-value = 0.313). Two studies^7,25^ fall outside the pseudo 95% confidence interval, providing further evidence of heterogeneity and bias.

## Discussion

Group B streptococci (GBS) are a major cause of neonatal sepsis, which results in one million neonatal deaths every year^1^. Also, studies have repeatedly shown that HIV-negative neonates that had been exposed to HIV during pregnancy are at increased risk for infectious morbidity and mortality (reviewed in^47^). In this systematic review and meta-analysis, we assessed the risk for GBS neonatal disease in HIV-exposed neonates. We also assessed the risk for rectovaginal GBS carriage in HIV-infected women, as rectovaginal carriage is a major risk factor for neonatal GBS disease.

We found convincing evidence that HIV-exposed neonates were at higher risk for GBS neonatal disease. When analyzing cases of early-onset GBS and late-onset GBS disease separately, we found that this increased risk pertains to late-onset disease only.

**Figure 6:**
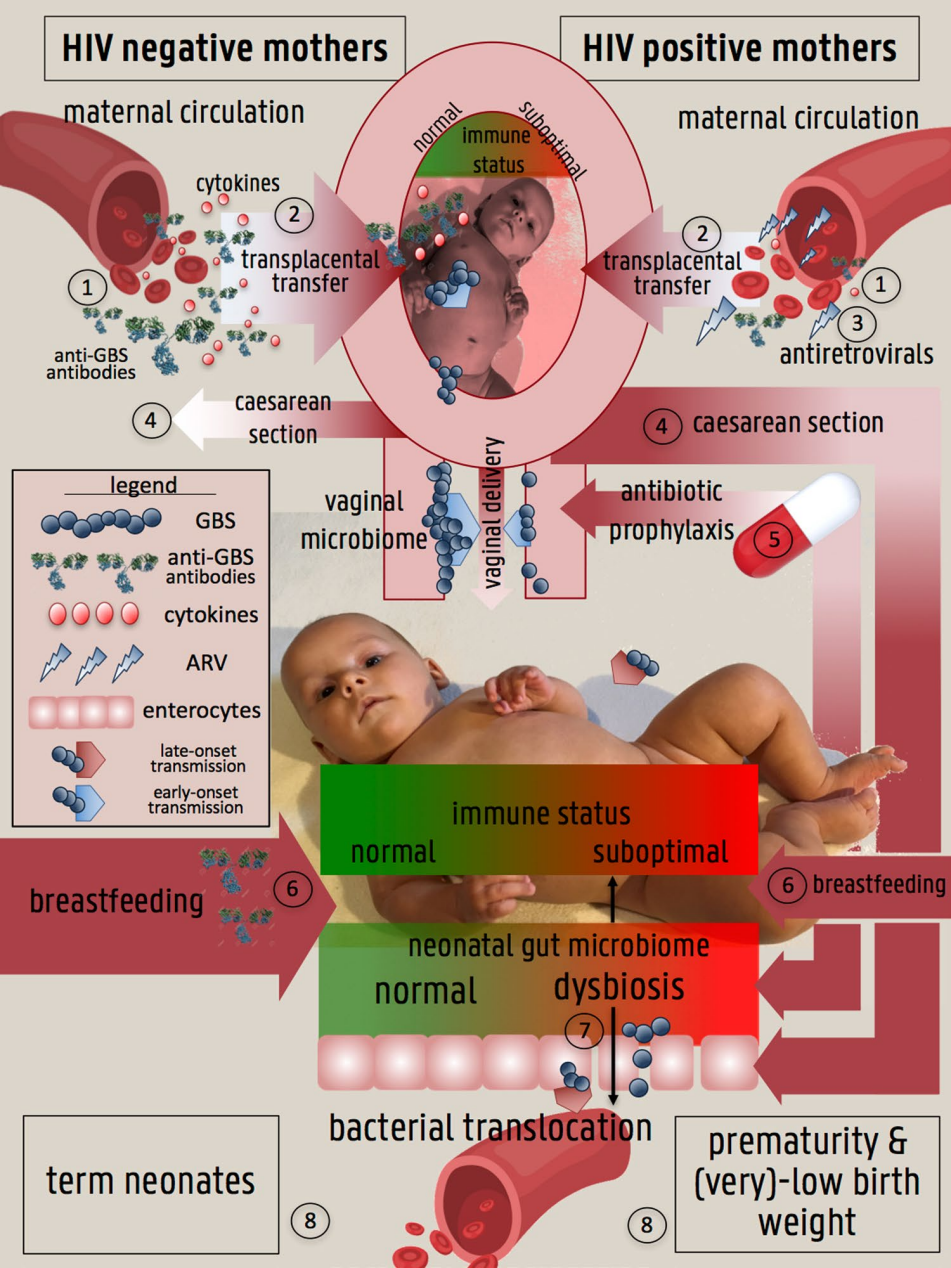
Framework explaining a higher prevalence of LOD (but not of EOD) in HIV-uninfected infants born to HIV + mothers compared to HIV- mothers. (**1**): lower concentrations of anti-GBS antibodies and cytokines in the circulation of HIV + mothers compared to HIV- mothers (↑EOD,↑LOD); (**2**): reduced transplacental transfer rate (symbolized by the narrower arrow) of anti-GBS antibodies and cytokines in HIV + mothers leading to reduced fetal immunity (↑EOD,↑LOD); (**3**): antiretrovirals in circulation of HIV + mothers might cause neonatal immune dysfunction (↑EOD,↑LOD); (**4**): higher rates of caesarean section in HIV + women compared to HIV- women (symbolized by the broader arrow), leading to a lower risk of GBS transmission during vaginal birth but also leading to neonatal gut dysbiosis (↓EOD,↑LOD); (**5**): antibiotic prophylaxis in HIV + women reduces vaginal carriages rates of GBS and hence risk GBS transmission during vaginal birth but is a contributor of neonatal gut dysbiosis (↓EOD,↑LOD); (**6**): lower prevalence of breastfeeding (symbolized by the narrower arrow) in HIV + mothers compared to HIV- mothers leading to a reduced passive immunity in their newborns and to neonatal gut dysbiosis (↑LOD); (**7**): neonatal gut dysbiosis leading to a reduced gut immunity and bacterial translocation (↑LOD); (**8**): HIV-infected women are at higher risk of having al low birth weight infant or a preterm delivery, both risk factors for EOD and LOD (↑EOD,↑LOD). ↓ = lower risk of, ↑ = higher risk of.

There are several factors associated with HIV infection in pregnant women or mothers that might explain why infants born to HIV-infected mothers are at increased risk for late-onset, but not early-onset GBS disease. In the case of early-onset GBS disease, the factors that increase or decrease the risk for early-onset GBS disease might be well-balanced, while in the case of late-onset GBS disease, the net result may be an enhanced risk (Fig. 6).

### Factors that might decrease early-onset GBS disease rates in HIV-exposed neonates

#### Antibiotic prophylaxis

HIV-infected pregnant women widely use antibiotic prophylaxis (co-trimoxazole), as recommended by the World Health Organization (WHO)^48^. Co-trimoxazole, largely effective against GBS^41^, is likely to be responsible for lower GBS rectovaginal carriage rates in HIV-infected pregnant women. For instance, we previously showed, using GBS-specific quantitative PCR (qPCR), that none of 30 Rwandan HIV-infected women, of which 93.3% were on antibiotic prohylaxis, carried GBS^49^. Seale and coworkers found that the rectovaginal GBS carriage rate was significantly less in 161 HIV-positive women on co-trimoxazole prophylaxis (n = 161, 3.1% GBS positive) compared to HIV-positive women not on prophylaxis (n = 239, 8.4% GBS positive) and compared to HIV-negative women (n = 7285, 12.1% GBS positive)^50^. As in GBS EOD, GBS is thought to be transmitted to the neonate from the vagina during passage through the birth channel or, more commonly, to the fetus *in utero* after breaking of the membranes by an ascending infection from the vagina, reduction of vaginal GBS carriage rates might reduce the risk for the early-onset form, but not for the late-onset form, where GBS is considered to be more frequently community- or hospital-acquired, or acquired through gut translocation. Antibiotic prophylaxis might also lead to neonatal gut dysbiosis: see further.

#### Caesarean delivery

The prevalence of EOD might be reduced by the practice of elective caesarean section. Elective caesarean section, i.e. caesarean delivery before labor and before rupture of membranes, has been shown to reduce the risk of mother-to-child transmission of HIV by 80% (60% to 70% of mother-to-child transmissions occur during labor and vaginal delivery^51,52^. This has led to dramatically increased ECS rates in HIV-infected women in some countries^52^. Because of the modes of transmission of GBS in EOD and LOD (see ‘antibiotic prophylaxis’), ECS might reduce the risk for EOD but not for LOD. Interestingly, Wu and coworkers found that of the 28 EOD cases, significantly more were born by vaginal delivery compared to cesarean delivery (64.3 versus 35.7%, respectively). Mehar and coworkers studied 141 cases of neonatal sepsis (61.3% EOD and 39.7% LOD). Of the neonates born by vaginal delivery, 38.1% became septic compared to 21.2% who were delivered by cesarean section^53^. Hook and colleagues^54^ compared pregnancy outcomes among 497 women undergoing elective repeat cesarean delivery to 492 who attempted trial of labor and 989 women with routine vaginal delivery. They found that rates of suspected and confirmed neonatal sepsis were significantly lower in the elective repeat cesarean delivery group, although absolute numbers of cases were very small (9, 26 and 26 cases of suspected sepsis in elective repeat cesarean delivery, trial of labor and vaginal delivery, respectively; and 0, 4 and 1 cases of confirmed sepsis in elective repeat cesarean delivery, trial of labor and vaginal delivery, respectively). Caesarean delivery might also lead to neonatal gut dysbiosis: see further.

### Factors that might increase late-onset GBS disease rates in HIV-exposed neonates

#### Formula feeding

Since 2009, the WHO recommends HIV-positive mothers to practice exclusive breastfeeding for their infants in combination with antiretroviral therapy (ART) for mother or infant. However, compared to HIV-negative women, HIV-positive women have been shown to preferentially choose exclusive replacement feeding or mixed feeding, or stop breastfeeding early, because of fear of HIV transmission through breast milk. This is a consequence of previous WHO guidelines recommending total avoidance of breastfeeding and/or because many advocates of programs for prevention of mother-to-child transmission recommend early weaning^55–61^. Lack of breastfeeding might hamper passive protection of the neonate against GBS neonatal disease as GBS specific IgA, present in breast milk, could offer GBS-specific protection^62^. Furthermore, HIV-infected mothers might have reduced IgA in breast milk, as has been shown to be the case for saliva^63^. These altered infant feeding practices are likely to have more effect on the incidence of LOD, considering the fact that most infants with EOD are already septic at birth or become septic shortly after birth.

Many other bioactive factors in breastmilk, such as growth factors and prebiotics, may contribute to a better immune response and development and these children are likely more resistant to infectious diseases^64^. This has been exemplified by some trials, where breastfed children born to HIV-positive mothers has significantly lower mortality rates compared to formula-fed children^65,66^. Formula feeding might also lead to neonatal gut dysbiosis: see below.

#### Neonatal gut dysbiosis

The human gut microbiome is involved in many aspects of health and disease by exerting metabolic, nutritional, and immunological effects on the host^67^. In healthy, vaginally delivered and term newborns, pioneering bacteria from the mother are encountered, tolerated and regulated by the naïve neonatal gut immune system. In turn, this emerging microbiome sculpts the gut immune, metabolic and barrier function^67,68^. This dynamic process is readily disrupted. Such disruption, termed dysbiosis, appears to be pivotal in development of LOS^67,69,70^. Neonatal gut dysbiosis compromises the integrity of the intestinal barrier and the development of the immune system, leading to an increased risk for bacterial gut translocation into the bloodstream of the neonates^69,71,72^.

The most important disturbing factors of the neonatal gut microbiome appear to be (i) C-section delivery, (ii) perinatal maternal and/or neonatal antibiotic use, and (iii) formula feeding^73^.

(i) The gut microbiome differences between neonates born by vaginal delivery and caesarean section are striking. A landmark study by Dominquez-Bello and coworkers found that the former harbored a gut microbiome closely resembling their mother’s vaginal bacteria (e.g. lactobacilli), while the latter harbored a gut microbiome resembling that of the maternal skin (e.g. *Staphylococcus*, *Corynebacterium*, *Propionibacterium* spp.)^74^. Other studies have confirmed that neonates born by caesarean section have disturbed gut microbiomes compared to neonates born by vaginal delivery (reviewed in^75^).
(ii) Multiple studies have shown that peripartum antibiotics disrupt the natural neonatal microbiome assembly^73^. For example, intrapartum antibiotics to prevent neonatal GBS disease has been associated with decreased bacterial diversity of the meconium^76^ and lower abundance of lactobacilli and bifidobacteria in the neonatal gut^73^. Administration of antibiotics to the neonate directly after birth has been associated with gut dysbiosis^73^ and with LOS^77^.
(iii) Formula feeding can cause perturbations of the neonatal gut microbiome compared to breast milk, even if given in small amounts during breastfeeding^73^. Breast milk contains a mix of nutrients, bacteria, antimicrobial proteins and secretory IgA that affects the shaping of the normal neonatal gut microbiome. Formula milk has for example been associated with a decreased prevalence of bifidobacteria^78^, thought to suppress the growth of potentially pathogenic bacteria^79^. Clinical studies have shown that lack of breastfeeding is a risk factor for neonatal LOD and meningitis^80–82^, and it is tempting to hypothesize that the mechanistic pathway explaining these findings is a combination of the above-discussed consequences of formula feeding/lack of breastfeeding, ie. a reduced passive immunity and a dysbiotic gut microbiome.

The risk of bacterial translocation causing LOS is exacerbated in preterms and neonates with (very)-low birth weight: these are not only at higher risk of the above-mentioned risk factors leading to gut microbiome dysbiosis, but also have an increased intestinal permeability and an immature immune system^79^.

### Factors that might increase EOD and LOD GBS disease rates in HIV-exposed neonates

#### Compromised immunity

Low maternal GBS-specific antibody concentrations have been shown to be correlated with susceptibility of neonates for GBS EOD and LOD^83–85^. Some important observations show that these protective antibodies are reduced in HIV-exposed neonates and HIV-infected mothers. Significantly lower GBS-specific antibody concentrations were measured in maternal/cord blood from HIV-infected mothers and neonates born from HIV-infected mothers^34,86^. Furthermore, a reduced transplacental transfer of GBS-specific antibodies has been shown in HIV-infected mothers^34,86^. In addition, the neonatal anti-GBS antibodies remained significantly lower at 16 weeks^86^. Le Doare and co-workers demonstrated a significantly lower antibody-mediated C3b/iC3b deposition on all investigated GBS serotypes (Ia, Ib, II, III and V) in HIV-infected women and neonates born to HIV-infected women, suggesting a reduced opsonophagocytotic immune effector function^86^. A recent phase 2 GBS vaccination trial (with a trivalent vaccine consisting of conjugated capsular polysaccharides of GBS serotypes Ia, Ib and III) in African HIV-positive and HIV-negative pregnant women showed that the vaccine was less immunogenic in HIV-infected women, irrespective of their CD4 count^87^.

Other perturbations of the immune system of HIV-infected women (even in the absence of overt immune dysfunction) might also play an important role. HIV-infected women were shown to have reduced transplacental transfer of hematopoietic cytokines. This may result in lower thymic output of CD4^+^ cells in their infants and a delay in immune cell maturation^17^. Furthermore, intrauterine exposure to HIV or its soluble products appears to cause a disturbed thymic function in HIV-uninfected newborns^88^.

#### Anti-retroviral therapy (ART)

ART, used to prevent mother-to-child transmission of HIV, might play a role as well. Nucleoside reverse transcriptase inhibitors (NRTIs) crossing the placenta could cause mitochondrial dysfunction in HIV-exposed uninfected infants, explaining a decrease in neutrophils, total lymphocytes, CD4^+^ and CD8^+^ cells counts^17^.

#### Prematurity and (very)-low birth weight

Prematurity and (very)-low birth weight are more prevalent in neonates born to HIV-infected mothers (reviewed in^89^) and both are risk factors for GBS EOD and GBS LOD^90–95^.

### Factors that might increase or decrease early-onset GBS disease rates in HIV-exposed neonates: meta-analysis of the risk for rectovaginal GBS carriage in HIV-infected women

Because rectovaginal GBS carriage is a major risk factor for early-onset neonatal GBS disease, we also assessed the risk for rectovaginal GBS carriage in HIV-infected women.

Our meta-analysis, based on 13 studies, did not show an association between HIV infection and rectovaginal GBS carriage, but most of the studies suffered from major limitations and we consider the currently available evidence weak and call for further study.

Few studies had adequate statistical power and few were designed to assess the association between HIV infection and rectovaginal GBS carriage. Most studies were at medium or high risk of bias related to representativeness of study groups, comparability of study groups, and/or outcome assessment.

Heterogeneity was considerable. Technical biases, such as the specimen collection and culture methods are likely to contribute substantially to this heterogeneity. For example, rectovaginal sampling has been shown to yield significantly higher recovery rates compared to vaginal sampling alone^96,97^. Furthermore, the use of a selective enrichment broth has been shown to improve GBS detection substantially^96,98^. However, rectovaginal sampling and the use of a selective enrichment broth, both recommended by the CDC, were used by only five studies. Also, the different study populations and eligibility criteria are likely to contribute to the observed heterogeneity.

Some important possible confounding factors were not taken into account. First, as described above, the use of antibiotic prophylaxis in HIV positive women, recommended by the WHO^48^, might be responsible for lower GBS rectovaginal carriage rates in HIV-infected pregnant women but was only taken into account in few studies. Secondly, none of the studies corrected for bacterial vaginosis (BV), a disturbance of the vaginal microbiome characterised by the deprivation of lactobacilli and the presence of anaerobes such as *Gardnerella vaginalis* and *Atopobium vaginae*. BV has been shown to be a risk factor for the acquisition of HIV^99^ but also to be negatively associated with vaginal GBS carriage^49,100^. Although speculatively, this might mask an association between HIV and GBS.

Finally, risk factors for early-onset GBS disease such as urinary tract infections and intrapartum fever have been reported to be more prevalent in HIV-infected women but were considered only by Cutland *et al*.^35^.

As the risk for EOD increases with heavier vaginal GBS colonization at the onset of labor or rupture of membranes^101^, future studies should also include quantitative molecular tests such as GBS-specific qPCR to determine the vaginal GBS load in relation to the HIV status. Furthermore, including the assessment of GBS serotype/genotype distribution in HIV-positive and –negative women will document whether more virulent GBS strains are present in HIV-positive women.

In conclusion, the evidence that HIV-exposed infants are at increased risk for late-onset GBS neonatal disease is accumulating. A compromised immune system, a neonatal gut microbiome dysbiosis, a lesser tendency to breastfeed but higher tendency for cesarean section and the use of prophylactic antibiotics in HIV-positive women might partly explain these findings. Public health interventions to prevent LOD are urgently needed in neonates born to HIV-infected mothers, even if themselves uninfected, and promoting feeding strategies such as breastfeeding or the use of probiotics and prebiotics have been shown to be exciting options to prevent^69,81,82,102^.

While we did not find evidence for an increased risk of rectovaginal GBS colonization in HIV-infected women, most studies conducted to date suffered major limitations. Further well-designed studies incorporating sensitive, quantitative (and serotype specific) GBS detection methods may further clarify the relation between HIV-positivity and GBS carriage.

## Electronic supplementary material

Supplementary Information
