# Supplementary material for: Role of HIV exposure and infection in relation to neonatal GBS disease and rectovaginal GBS carriage: a systematic review and meta-analysis
Source: Sci Rep. 2017 Oct 23;7:13820. doi: 10.1038/s41598-017-13218-1 (PMC5653843; doi:10.1038/s41598-017-13218-1)
Supplement: Supplementary file 1 — Supplementary Information [file 41598_2017_13218_MOESM1_ESM.pdf]

Role of HIV exposure and infection in relation to neonatal GBS disease and rectovaginal GBS carriage: a systematic review and meta-analysis.

Piet Cools[1*], Janneke H.H.M. van de Wijgert[2], Vicky Jespers[3], Tania Crucitti[4], Eduard J. Sanders[5,6], Hans Verstraelen[7], Mario Vaneechoutte[1].

[1] Laboratory Bacteriology Research, Department of Microbiology, Immunology and Clinical Chemistry, Faculty of Medicine and Health Sciences, Ghent University, De Pintelaan 185, Ghent, Belgium; [2] Department of Clinical Infection, Microbiology and Immunology, Institute of Infection and Global Health, University of Liverpool, Brownlow Hill, Liverpool, UK; [3] HIV and Sexual Health Group, Department of Public Health, Institute of Tropical Medicine, Nationalestraat 155, Antwerp, Belgium; [4] HIV/STI Reference Laboratory, Department of Clinical Sciences, Institute of Tropical Medicine, Nationalestraat 155, Antwerp, Belgium; [5] Centre for Geographic Medicine Research - Coast, Kenya Medical Research Institute (KEMRI), PO Box 230-80108, Kilifi, Kenya; [6] Nuffield Department of Medicine, University of Oxford; [7] Department of Obstetrics and Gynaecology, Vulvovaginal Disease Clinic, Ghent University Hospital, De Pintelaan 185, Ghent, Belgium

**Studies without reports of predefined outcomes or exposures (32)**
Al-Harthi L, 1999; Alikor DE, 2006; Bahar MA, 2000; Bakr AF, 2005; Barug D, 2014; Beigi RH, 2004; Berkley, 2005; Brouwer MC, 2013; Charles NC, 2014; Chihana ML, 2014; Demba, 2005; Downing M, 2006; Duke T, 1999; Farley MM, 1993; Galiwango E, 2015; Gichuhi S, 2009; Gwee A, 2012; Hassanzadeh P, 2011; Kong JC, 2012; Koumans EHA, 2012; Le Doare K, 2015; Martin R, 1997; Moyes J, 2013; Mulu W, 2015; Mussi-Pinhata MM, 2007; Nicaise C, 2003; Nielsen HU, 2002; Pameh W, 2002; Robertson AJF, 2012; Schuchat A, 2002; Weisbord JS, 2001; Weldearegawi B, 2013
**Book chapters, reviews (7)**
Althaus FA, 1991; Baltimore RS, 2003; Bhutta ZA, 1997; Duke T, 2005; Khare MM, 2005; Kind C, 1989; Shann, 2011
**Studies without an HIV-negative or GBS-negative control group (9)**
Arrive E, 2010; El Beitune P, 2007a; El Beitune P, 2007b; El Beitune P, 2006; Joao EC, 2011; Joao 2013; Sagna T, 2010; Sonnex C, 2013; Wiggins H, 2014
**Studies without reported measure of effect or when measure of the effect could not be calculated (11)**
Cherpes TL, 2003; Elliott B, 1990; Lito D, 2013; Schrag SJ, 2003; Moyo SR, 1995; Nathoo KJ, 1999; Benedetto C, 2004; Buscemi L, 2004; Ruffini E, 2014; Schuetz, AN, 2011; Top KA, 2012
**Manuscripts reporting previously published data (4)**
Adler C, 2015; Epalza C, 2009; Goetghebuer T, 2012; Ronge R, 2007

Supplementary Table 1: Studies excluded after full text evaluation.
The number of studies is indicated between brackets. Ali F, 1996 was not found.

**Selection**	
**1 Representativeness of the exposed group**	
a Truly representative of the average women *(neonates)* in the community	*
b Somewhat representative of the average women *(neonates)* in the community	*
c Selected group of women *(neonates)*	
d No description of the sampling strategy	
**2 Representativeness of the non-exposed group**	
a Drawn from the same community as the exposed group	*
b Drawn from a different source	
c No description of the derivation of the non-exposed group	
**3 Sample size (total number of cases analyzed)**	
a Justified and satisfactory	*
b Not justified or not satisfactory	
**4 Ascertainment of the HIV infection *(exposure)***	
a Validated assay performed for the study or through medical records	*
b Self-reported without further documentation	
**Comparability**	
**The subjects in different comparison groups are comparable, based on the study design or analysis**	
a Controlling for confounding factors (logistic regression or propensity scoring)	**
b Subjects in different groups are comparable	*
c No reporting on comparability between comparison groups and no control for confounding	
**Outcome**	
**Assessment of the outcome (rectovaginal GBS carriage)**	
a Sampling, culturing, and identification according to CDC[#] guidelines	**
b Culturing and identification according to CDC guidelines	*
c Sampling, culturing, and identification not adequately performed/described	
d No description	
**Assessment of the outcome (GBS neonatal disease):**	
a Blood samples, cerebrospinal fluid (CSF), or other normal sterile site; BactAlert and/or GBS specific latex agglutination on CSF	**
b Culture methods not described	

Risk of bias	
**Selection**	4 stars, low risk of bias; 2 or 3 stars, medium risk of bias; 0 or 1 star(s), high risk of bias
**Comparability**	2 stars, low risk of bias; 1 star, medium risk of bias; 0 stars, high risk of bias
**Outcome**	2 stars, low risk of bias; 1 star, medium risk of bias; 0 stars, high risk of bias

Supplementary Table 2: Quality appraisal tool: adjusted Newcastle-Ottawa Quality Assessment Scale.
#CDC, Centers for Disease Control and Prevention, Bethesda, Maryland.

Author, year	Country	Study design	Study period	Study setting and population	HIV exposure assesment	Outcome assessment and EOS/LOS definition	Correction confounding
Cutland, 2012	SA	XS	2004-2007	Infants born at a public secondary-tertiary level of care hospital	Medical records, active voluntary HIV testing programme for pregnant women (in the community, >96% of pregnant women are tested (PC), in parent study, 99% were tested)	EOS, 0-3 d; LOS, 4-28 d. Active surveillance of neonatal/pediatric wards to ID study neonates, abstraction information related to neonatal sepsis from medical records. **1**. B, samples other sterile sites **2.** B: BacT/Alert, other samples: routine methods **3**. Routine methods	PSM (EOS only)
Cutland, 2015	SA	R	2004-2008	Infants born at a public secondary-tertiary level of care hospital	Medical records, HIV ELISA results from maternal blood tests (>96% of pregnant women accept testing (PC). Sensitivity analysis of incidence accounted for GBS case-patients with unknown maternal HIV status	GBS disease, 0-90 d. Cases identified through screening of ward admissions and microbiological records within 24h of GBS ID. **1.** B, CSF. **2.** BacT/Alert	None
Dangor, 2015[a]	SA	XS	2013	Tertiary-level care hospital serving black-African community of Soweto and surrounding areas. I, >= 2500 g.	Medical records, pregnant women HIV tested by RT, CON ELISA	GBS disease, <90 d; EOS, < 7 d; LOS, 7–89 d. GBS cultured from blood, CSF or normally sterile sites, or GBS in CSF by LA. **1.** B, CSF **2.** B positive on BacT/Alert and CSF: BA or CA, BHIB (CSF), AER, CO_2, 35°C, 72h. **3.** direct on CSF: LA, GS. Colonies ID: CAT, BEA, ESC, CAMP, GBALA (all PC)	None
Dangor, 2015[b]	SA	CC	2012-2014	Infants with GBS disease recruited from three secondary-tertiary care public hospitals; EOD controls from one public hospital; LOD controls identified through the birth registries	HIV testing of pregnant women routinely performed and confirmed using two independent RT	GBS disease, <90 d; EOS, < 7 d; LOS, 7–89 d. GBS cultured from blood, CSF or normally sterile sites, or GBS in CSF by LA. **1.** B, CSF **2.** B positive on BacT/Alert and CSF: BA or CA, BHIB (CSF), AER, CO_2, 35°C, 72h. **3.** direct on CSF: LA, GS. Colonies ID: CAT, BEA, ESC, CAMP, GBALA.	None
Epalza, 2010	B	R	2001-2008	Hospital with tertiary neonatal center and HIV referral center in an urban setting. Most infants born from mothers of SSA origin living in poor socioeconomic conditions	Review of medical records, all pregnant women are tested for HIV (ELISA, CON WB)(PC)	EOS, 0-6 d; LOS, 7-90 d. **1.** B, CSF (PC) 2. BacT/Alert (PC)	None
Frigati, 2014	SA	R	2010-2011	Infants born at a primary and a tertiary level of care hospital	Medical records	GBS disease, 0-90 d; EOS, 0-6 d; LOS, 7-90 d. Cases identified throught the hospital database. **1.** B, CSF **2.** BacT/Alert	None

Supplementary Table 3: Digest of included studies reporting HIV exposure of neonates and GBS neonatal disease.

Author: [a], Dangor, 2015, JID; [b], Dangor, 2015, Plos ONE

Country: B, Belgium; SA, South-Africa

Study design: CC, case-control; R, retrospective; XS, cross-sectional

Study setting and population: EOD, early-onset disease; I, inclusion criteria; LOD, late-onset disease; SSA, sub-Saharan Africa

Exposure assessment: CON, confirmatory test; RT, rapid test; S, serology; WB, Western blotting; PC, personal communication

Outcome assessment: EOD, early-onset disease; LOD, late-onset disease; d, days; PC, personal communication

1. *Sampling and transport medium*: B, blood sample; CSF, cerebrospinal fluid

2. *Culture conditions*: AER, aerobic conditions; BA, blood agar; BHIB, brain heart infusion broth; CA, chocolate agar

3. *Identification:* BEA, growth on bile esculin agar; CAMP, Christie, Atkins, and Munch-Petersen test; CAT, catalase test; ESC, esculine hydrolase test; GS, Gram stain; LA, GBS specific latex agglutination

Author, year	Country	Study design	Study period	Study setting and population	Age (years)	HIV exposure assesment	Outcome assesment	Correction confounding
Cutland, 2012	SA	XS	2004-2007	Pregnant women presenting in labor at a secondary-tertiary level of care hospital	Mdn, 26; R, 12-51	Medical records, active voluntary HIV testing programme for pregnant women (in the community, >96% accept testing (PC))	**1.** LV **2.** A; HBA +/- C (10 µg/mL) and NA (15 µg/mL); TH+G (8 µg/mL) and NA (15 µg/mL), HBA. **3.** GPBHC: CAMP, ESC, LA.	PSM
Dangor, 2015[a]	SA	XS	2013	Pregnant women delivering at a tertiary-level care hospital serving black-African community of Soweto and surrounding areas.	R, 18.2-42.7	Medical records, pregnant women HIV tested by RT, CON ELISA	**1.** LV, R. **2.** A w/o charcoal; CHA; 37 °C; 18–24-(48)h; AER. **3.** GBS-like colonies: CAMP; ESC, CAT, LA	None
Djigma, 2011	BF	XS	2009	Non-pregnant women seeking gynaecological assistance	HIV+: Mdn, 33.0; R, 28-44; HIV-: Mdn, 35.0; R, 27-45	Questionnaire	**1.** V. **2.** CA; 37 °C; 24h **3.** mini-galleries	None
Ernest, 2015	T	XS	2011-2012	Pregnant women (GA, 28-42 w) attending consultant and teaching hospital, E,: ab within 2 w before recruitment	M 25.6 +/- 0.31; R, 14-39	Questionnaire	**1.** LV, PA **2.** SBA, 37 °C, CO_2, 24 h. GPBHC, CAT negative, SC SBA + SXT and BAC discs. 3. Confirmation CAMP, LA	None
El Beitune, 2006	Br	XS	2002-2004	Pregnant women (GA, 35-37 w), singleton gestation; E, hepatitis B, hepatitis C, syphilis, previous history of a newborn with GBS EOD.	R, 16-43	2 ELISAs, CON WB	**1.** LV, A **2.** TH + 8 mg/ml G and 15 mg/ml NA (transport), +250 ul SB, 35 °C, 18 h; BA + G and NA (idem conc), 35 °C, 48h. **3**. (GP)BHC, LA	None
Gray, 2011	M	XS	2008-2010	Pregnant women (GA, 3th trimester) recruited from the labor ward of hospital	>= 16; M, 25.2 +/- 5.9	2 RT (Detemine™, Uni-Gold™), discordant results: 3rd RT (SD-Bioline)	**1.** LV, R **2.** TH+ 15 ug/ml NA and 10 ug/ml C; 18-24 h. **3.** Phenotypic characteristics, CAMP, LA	LR
Joachim, 2011	T	XS	2008-2009	Pregnant women (GA, >= 37 w) attending antenatal clinics of a tertiary facility which handles referrals from peripheral and upcountry hospitals for routine antenatal visits; E, ab within 2 weeks prior to recruitment	M, 26.6 +/- 5.1; R, 16-44	RT (SD-Bioline HIV 1/2), if negative: HIV status negative, if reactive: CON 2nd RT (Determine™). HIV status positive if both tests positive	**1.** HV, R **2.** TH (transport), TH + NA (15 mg/L) and G (8 mg/L), 37°C, 5% CO_2, 24 h-(48h), SC on SBA. **3.** Presumptive ID: GS, CAT, HAEM, HIP, CAMP, LA; Confirmation, LA.	None
Mavenyengwa, 2010	Z	XS	2003-2005	Pregnant women (GA, +/- 20 w) attending antenatal clinic and recruited consecutively at three centres (rural, an urban, and a mixed rural-urban).	R, 16-45	RT (HIV Ag/Ab Combo). CON WB. Interpretation according to American Red Cross guidelines.	**1.** LV, R **2.** S, CAN agar base with SB, and in TH+C (10 mg/L) and NA 15 mg/L. (Lim??), 37 °C, 24 h-(48), looking for b-haemolytic or nonhemolytic streptococcal colonies typical of GBS. TH was sub-cultured onto CAN blood agar plates before 24 h incubation at 37 °C.	None
Mitima, 2014	DRC	XS	2012-2013	Pregnant women, (GA, 3th trimester), attending prenatal care in 12 healthcare centers. Women mostly of low socio-economic status, urban population. E, women with fistulae, women currently on ab.	10%, <18; 66%, R, 18-35; 24% >35	Medical records; RT (Determine), CON 2nd RT (Unigold or Double Check Gold). Positive if 2 RT positive.	**1.** V **2.** A, BA + NA (15 mg/mL) and C (10 µg/mL); 37°C, CO_2, 48 h **3.** BHC: GS, CAT, HIP, CAMP.	None
Sebitloane, 2011	SA	XS	2 years	Pregnant women (GA, >= 36 w) in whom vaginal delivery was anticipated	>= 18	RT (Determine[TM] HIV1/2), CON 2nd RT (SmartCheck).	**1.** V **2.** A, CEF CLED, BAC CA, and laked blood CEF	None
Shah, 2011	US	R	1997-2007	Ethically diverse pregnant women attending prenatal care in an urban public hospital	HIV+: M, 29.5 +/- 5.8; HIV-: M, 28.0 +/- 6.3	HIV status abstracted from medical records, HIV test not mentioned.	Medical records. : defined as having a positive rectovaginal swab recovered within 5 weeks of delivery or GBS bacteriuria at any point during index pregnancy, as per the 2002 guidelines CDC	LR
Temmerman, 1992	K	XS	1989-1990	Pregnant women with clinical signs and symptoms of spontaneous abortion (GA, < 20 w) and pregnant women (GA, >20 w) visiting a non-private referral university	M, 25.9	EIA (2X), CON WB	**1.** HV **2.** CAN CA+SB	None

				hospital. E, women on ab 24h before visit, women with induced or clinically septic abortion or with axillary temp >38°C, severe anemia, shock.				
Ulla, 1993	S	XS	1986-1991	Female sex workers recruited at a family planning center	M, 25; R, 18-50	ELISA, CON WB	**1.** V **2.** TSA-HB	None

Supplementary Table 4: Digest of studies reporting HIV infection of women and rectovaginal GBS carriage.

Author: [a], Dangor, 2015, JID

Country: Br, Brazil; BF, Burkina Faso; DRC, Democratic Republic Congo; K, Kenya; M, Malawi; S, Spain; SA, South-Africa; T, Tanzania; US, United States; Z, Zimbabwe;

Study design: R, retrospective; XS, cross-sectional

Age: M, mean; Mdn, median; R, range

Study setting and population: E, exclusion criteria; GA, gestation age; I, inclusion criteria; w, weeks

Exposure assessment: HEU, HIV-exposed uninfected; 2X, positive samples repeated with same method; CON, confirmatory test; RT, rapid test; S, serology; WB, Western blotting; PC, personal communication

Outcome assessment:

1. *Sampling and transport medium*: A, anal swab; B, blood sample; CSF, cerebrospinal fluid; HV, high vaginal swab; LV, low vaginal swab; PA, perianal swab; R, rectal swab; RV, rectovaginal swabs; V, vaginal swab;

2. *Culture conditions*: A, Amies transport medium; AER, aerobic conditions; BA, blood agar; BAC, bacitracin; BHIB, brain heart infusion broth; C, colistin; CA, chocolate agar; CA+SB, Columbia agar + 5% sheep blood; CAN, colistin nalidixic acid agar; CHA, CHROMagar StrepB; CEF CLED, cefoxitine CLED plates; G, gentamycin; HBA, 5% horse blood agar; NA, nalidixic acid; S, Stuart's transport medium; SB, sheep blood; SBA, 5% sheep blood agar; SC, subcultures; TH, Todd Hewitt broth; TSA-HB, tryptic soy agar + 5% human blood; (-48h), if no growth after 24h, another day was incubated

3. Identification: BEA, growth on bile esculin agar; CAMP, Christie, Atkins, and Munch-Petersen test; CAT, catalase test; ESC, esculine test; GS, Gram stain; (GP)BHC, (Gram-positive) β-haemolytic colonies; HAEM, haemolytic activity; HIP, hippurate test; LA, latex agglutination; PC, personal communication

Study	Selection				Comparability	Outcome
	Representativeness exposed group	Representativeness non-exposed group	Total number cases analyzed	Ascertainment HIV infection/ exposure		
**HIV infection in women and rectovaginal GBS carriage**						
Cutland, 2012	*	*	*	*	**	*
Dangor, 2015[a]		*		*	*	*
Djigma, 2011		*				
Ernest, 2015	*	*			*	
El Beitune, 2006			*	*		**
Gray, 2011	*	*	*	*	**	**
Joachim, 2011	*	*		*	*	**
Mavenyengwa, 2010		*		*		**
Mitima, 2014	*	*		*		
Sebitloane, 2011	*	*		*	*	
Shah, 2011				*	**	**
Temmerman, 1992		*		*		
Ulla, 1993		*		*		
**HIV exposure neonates and GBS neonatal disease**						
Cutland, 2012	*	*	*	*	**	**
Cutland, 2015	*	*	*	*	*	**
Dangor, 2015[a]	*	*	*	*	*	*
Dangor, 2015[b]	*	*	*	*	**	**
Epalza, 2010	*	*	*	*		*
Frigati, 2014	*	*	*	*		**

Supplementary Table 5: Critical appraisal of the included studies.

[a], Dangor, 2015, JID; [b], Dangor, 2015 PONE
